# Examining Individual Differences in Singing, Musical and Tone Language Ability in Adolescents and Young Adults with Dyslexia

**DOI:** 10.3390/brainsci12060744

**Published:** 2022-06-06

**Authors:** Markus Christiner, Bettina L. Serrallach, Jan Benner, Valdis Bernhofs, Peter Schneider, Julia Renner, Sabine Sommer-Lolei, Christine Groß

**Affiliations:** 1 Centre for Systematic Musicology, Faculty of Arts and Humanities, University of Graz, Glacisstraße 27, 8010 Graz, Austria; p.schneider.hd@web.de; 2 Jazeps Vitols Latvian Academy of Music, K. Barona Street 1, LV-1050 Riga, Latvia; valdis.bernhofs@jvlma.lv (V.B.); christine.michaela.gross@jvlma.lv (C.G.); 3 Department of Neuroradiology and Section of Biomagnetism, University of Heidelberg Medical School, University of Heidelberg, INF 400, 69120 Heidelberg, Germany; bxserral@texaschildrens.org (B.L.S.); benner@musicandbrain.de (J.B.); 4 Edward B. Singleton Department of Radiology, Texas Children’s Hospital and Baylor College of Medicine, Houston, TX 77030, USA; 5 Department of East Asian Studies, University of Vienna, Sensengasse 3a, 1090 Vienna, Austria; julia.renner@univie.ac.at; 6 Department of Linguistics, University of Vienna, Sensengasse 3a, 1090 Vienna, Austria; 7 Austrian Academy of Sciences, Doktor-Ignaz Seipel-Platz 2, 1010 Vienna, Austria; sabine.sommer-lolei@univie.ac.at

**Keywords:** dyslexia, singing, musical ability, P1, N1, and P2 latencies, Mandarin, sound-symbol correspondence, pronunciation, magnetencephalography, frequency, language ability

## Abstract

In recent years, evidence has been provided that individuals with dyslexia show alterations in the anatomy and function of the auditory cortex. Dyslexia is considered to be a learning disability that affects the development of music and language capacity. We set out to test adolescents and young adults with dyslexia and controls (N = 52) for their neurophysiological differences by investigating the auditory evoked P1–N1–P2 complex. In addition, we assessed their ability in Mandarin, in singing, their musical talent and their individual differences in elementary auditory skills. A discriminant analysis of magnetencephalography (MEG) revealed that individuals with dyslexia showed prolonged latencies in P1, N1, and P2 responses. A correlational analysis between MEG and behavioral variables revealed that Mandarin syllable tone recognition, singing ability and musical aptitude (AMMA) correlated with P1, N1, and P2 latencies, respectively, while Mandarin pronunciation was only associated with N1 latency. The main findings of this study indicate that the earlier P1, N1, and P2 latencies, the better is the singing, the musical aptitude, and the ability to link Mandarin syllable tones to their corresponding syllables. We suggest that this study provides additional evidence that dyslexia can be understood as an auditory and sensory processing deficit.

## 1. Introduction

Dyslexia is one of the most common neurodevelopmental disorders in children and adolescents, with a worldwide prevalence ranging between 5–10% [1,2]. Across languages, individuals with dyslexia have learning impairments leading to lower language and music performance. Regarding language performance, dyslexia is often associated with lower phonological, spelling, decoding, and word-recognition skills [3,4,5]. In addition, dyslexics show several deficits in musical performance [3,6,7,8]. Impairments were found in both the discrimination of elementary and complex auditory sounds [8], as well as in musical performance [7]. Compared to controls, individuals with dyslexia performed worse when introduced to singing or playing musical instruments [7]. There is growing evidence that lower musical perceptual ability, and lower musical and language performance in dyslexics, is associated with the altered anatomy and function of the auditory cortex [3,8,9]. Therefore, particular attention has been paid to the auditory evoked P1–N1–P2 complex to study individual differences in auditory processing [3,10,11,12,13,14,15]. 

While dyslexics show deficits in music and language ability, musicians or individuals with high musical talent are on the other side of the spectrum. Musical training enhances auditory perception accuracy [16,17,18,19,20,21], which in turn is important for language development and capacity [21,22,23,24]. Studies on positive music-to-language transfer can be roughly subdivided into two main dimensions: studies using perceptual musical measurements and their comparison to language perception and performance tasks [25,26]; and studies using music performance measures (e.g., instrument playing and singing) or music-training measures and their comparison to language capacities including, among others, pronunciation [27,28,29,30,31] and reading [32]. Interestingly, accurate perception does not always predict accurate reproduction. Individuals performing well in perceiving foreign languages do not necessarily pronounce these utterances accurately [33]. The literature concerning the relationship between singing and pitch perception [34] is contradictory, with some studies reporting a relationship and others suggesting no link.

The speech perception of unfamiliar languages is perceived more precisely by musicians and by individuals with higher musical ability [28,35,36]. In this respect, several hypotheses have been put forward that focus on perceptual parameters with the objective to provide crucial information about the underlying dimensions as to why musical training improves language functions [37]. Patel’s OPERA hypothesis, for instance, proposes musical training to impose five conditions leading to adaptive plasticity in networks that are relevant for speech processing [37]. These are: overlap, precision, emotion, repetition, and attention. Two of them, namely, overlap and precision, are important for the acquisition of tone languages. Pitch discrimination ability in speech improves through musical training [38], which is why overlaps between music and language are rather salient in Mandarin, a tone language in which tones determine semantic information. The acquisition of the so-called tone syllables requires precise tonal ability. Mandarin has four different tones, which are generally referred to as ‘high level tone’, ‘(high) rising tone’, ‘falling-rising tone’, and ‘falling tone’ [39]. Mandarin native speakers are sensitive to tonal information in language, which provides a possible explanation why tone language speakers, compared to non-tone language speakers, possess enhanced pitch memory, higher tonal skills, and improved pitch processing ability [40]. This is in line with findings that musical training facilitates lexical tone identification [41] and the learning of Mandarin in general [42]. 

While there is a large body of literature on music and language functions, studies focusing on music production tasks such as singing and its relationship to language functions are limited. In general, there are two main branches of singing studies that focus on the positive transfer from singing to language: studies using singing as a tool to learn new vocabulary [43,44], and studies focusing on singing ability and its relationship to language ability. The latter used phonetic aptitude measures in which utterances of unfamiliar foreign languages had to be pronounced [36,45,46] or foreign accents had to be imitated [31] (so-called delayed mimicry paradigm [47]). These studies provide growing evidence that singing ability is particularly associated with the ability to mimic foreign accents and the ability to imitate and to pronounce foreign words and longer sequences of phrases [31,45,46,48,49]. In general, musical training is associated with the improved processing of acoustic input. Both singing ability and language pronunciation rely on vocal-motor skills and the integration of sensory and vocal-tract-related motor representations [27,28,35,45]. This has also been suggested to be a possible reason why singing ability is more strongly associated with language pronunciation than perceptual musical measures [28,35]. On the other hand, singing as a musical performance can be defined as a musical production task. The same is true when individuals are instructed to learn to pronounce new words in an unknown language. In addition, pronunciation tasks provide information about individual differences in phonological ability [31,36,45,46]. 

Another well-studied language component is phonemic coding ability. It describes the process in which sounds need to be retained and linked to a phonetic symbol. The ability to connect sounds and symbols is the fundamental basis for reading and writing processes. Research has shown that individuals diagnosed with dyslexia have particular difficulty with reading, spelling, and writing [4,50]. The *Precise Auditory Timing Hypothesis* (*PATH*) suggests that auditory musical training not only improves musical performance but also phonological awareness and phonemic coding ability [51]. Indeed, research has shown that musical training improves reading ability in first and foreign languages [32], and, in addition, it has been associated with phonemic awareness [52]. 

So far, our research has been mainly focused on music-related aspects, including the evaluation of differences in musical ability and on the auditory cortex of dyslexics compared to non-affected controls. As music and language share a large set of characteristics, our goal was to uncover distinct mechanisms for language measures in dyslexics and controls [8,9]. Therefore, we used our previously developed tone-language measures in Mandarin, in which the ‘high level tone’, the ‘(high) rising tone’, the ‘falling-rising tone’, and the ‘falling tone’ were embedded in Mandarin discrimination and Mandarin sound-syllable tone recognition tasks, and we also employed Mandarin pronunciation tasks of a former study [53]. In addition, we used previous research design in which music perception and music performance (singing) tasks were assessed. Our aim was to uncover whether we would also detect similar relationships between tone language performance and the auditory evoked responses than we found in previous studies which focused on musical capacities.

## 2. Materials and Methods

### 2.1. Participants

Fifty-two participants were included in this study, twenty six in each of the two groups (dyslexia and controls). Further information about the participants is contained in the Supplementary Materials (see Table S1). All the subjects were tested for their ability to discriminate and to detect tonal changes in paired Mandarin statements. We also assessed the participants’ ability to pronounce Mandarin and introduced measures that provide information about individual differences in elementary auditory skills, musical aptitude, and singing ability. None of the participants reported being able to speak or to comprehend Mandarin. All participants were native German speakers. The participants of this study were part of the larger combined cross-sectional and longitudinal research project “AMseL” (Audio- and Neuroplasticity of Musical Learning). This research has mainly focused on the impact of musical practice on cognition and the brain. The AMseL project was conducted at the University of Heidelberg (2009–2020) and founded by the German Federal Ministry of Education and Research (BMBF) and the German research foundation (DFG).

Dyslexics were diagnosed according to the Pediatric Neurology standards of the University Hospital Heidelberg, using the *Ein Leseverständnistest für Erst- bis Sechstklässler* (*ELFE 1-6*) [54] to assess spelling skills and the *Hamburger Schreib-Probe* (*H-LAD*) to assess phoneme discrimination [55]. All participants had normal hearing (defined as ≤20 dB HL pure-tone thresholds from 250 to 8000 Hz). Participants reported no comorbidities or history of neurological disorders.

### 2.2. Musical Background

The musical competence of each participant was assessed by obtaining the musical experience (questionnaire) and by calculating an index of cumulative musical practice (IMP). The IMP was defined as the product of the number of years of formal music education and the number of hours per week spent practicing a musical instrument [3,8,56]. However, compared to dyslexics (*M* = 7.30, *SE* = 1.55), the control group showed a tendency to higher musical experience (*M* = 9.67, *SE* = 1.20); the difference was statistically non-significant (*t*(50) = 1.21, *p* = 0.23; *r* = 0.17).

### 2.3. Measuring Mandarin Ability

Three Mandarin measures comprising tone discrimination, syllable recognition, and pronunciation tasks were developed to assess individual differences in Mandarin ability. The collection of our Mandarin measures included all four syllable tones: the ‘high level tone’ (5-5), the ‘(high) rising tone’ (3-5), the ‘falling-rising tone’ (2-1-4), and the ‘falling tone’ (5-1) [39]. However, we did not analyze tones separately, and we used scores like we used in a previous study [53]. 

#### 2.3.1. Tone Discrimination Task (Mandarin D)

The tone discrimination task consisted of 18 paired samples. The paired samples were either identical or contained a single tonal change in a particular syllable in the second statement (e.g., bùzhì versus bùzhī). The 18 samples comprised between two and eleven syllables. The first played statement was separated by a pause of 1 s. from the second statement. After listening to the statements, the participants had to indicate whether both statements were the same or different. Prior to performing the Mandarin D measure, the participants received a practice unit comprising 4 items (iterations permitted). After a short break of about five minutes, the participants had to take the test in a single run.

#### 2.3.2. Syllable Tone Recognition Task (Mandarin S)

Assessing individual differences in phonetic coding ability can be performed by using well-known language measures. For instance, the *Phonetic Script*, which is a subtest of the *Modern Language Aptitude Test* (*MLAT*) by Carroll and Sapon [57], or the more recently developed *LLAMA E* sound-symbol correspondence task by Meara [58] would be appropriate tools to evaluate individual differences in the ability link sounds to symbols. However, while the MLAT uses English as the target language, LLAMA is based on a British Columbian Indian language. To our best knowledge, there is no Mandarin sound-symbol correspondence task that is based on tone syllable recognition. Therefore, we developed a similar sound-symbol correspondence task in which we additionally embedded a tonal change of a tone syllable.

For the syllable tone recognition task, the participants had to listen to two paired samples and decide in which second statement syllable the tonal change occurred. The syllable tone recognition task consisted of 16 paired samples. Syllables were presented in visual form, and each syllable was indicated as a separate unit. As the paired statements only exhibited one tonal difference, the wording and syllable structure were equal for both statements, thereby only removing the diacritics. The word and phrase length of the samples varied between two and seven syllables. After completing a practice unit (repeated iterations permitted), participants took a five-minute break before they had to perform the tasks in a single session.

#### 2.3.3. Mandarin Pronunciation Task (Mandarin P)

The language production task consisted of nine Mandarin phrases (spoken by native speakers) of seven, nine, and eleven syllables. The subjects were instructed to repeat the phrases after listening to them for the third time. We used Mandarin samples that had been developed for assessing individual differences in Mandarin pronunciation in previous studies [36,45,46,53]. Recordings of the participants were normalized for their loudness and rated by Mandarin native speakers. Raters were instructed to evaluate how well participants globally imitated the Mandarin samples. Scores ranged between zero and ten. The interrater reliability was assessed by intra-class coefficient analysis, as provided in the Supplementary (see Table S2).

### 2.4. Gordon’s Musical Ability Test

The Advanced Measures of Musical Audiation (AMMA test) by Gordon [59] was used to assess the musical ability of the participants. It consists of rhythmic and tonal discrimination tasks with which the ability to internalize musical structures is assessed. The paired musical statements are embedded in one single test construction. Participants must indicate whether the paired statements are equal or different. In the different condition, participants additionally have to indicate whether the recognized difference was of tonal or rhythmical origin. The test consists of 33 items, including 3 familiarization/training tasks to start with. The score of all subtests which is generated is indicated by AMMA total in this study.

### 2.5. Tone Frequency and Duration Test

To assess individual differences in basic sound discrimination abilities, we used two subtests of the primary auditory threshold measure KLAWA (Klangwahrnehmung): tone frequency and duration. Tone frequency measures the ability to discriminate between high and low tones, while duration provides information about individual differences in distinguishing between short and long tones. KLAWA is an inhouse computer-based threshold measure. Difference limes are measured for tone frequency (“low vs. high”) and duration (“short vs. long”). Based on an “alternative-forced choice” [60], we used these measurements for assessing individual perceptual thresholds to study auditory processing (cent = 1/100 semitone for recording the pitch, and milliseconds (ms) for time measurements). In a forced-choice paradigm, reference and test tones (sinusoids), separated by an inter-stimulus interval of 500 ms, were presented. Participants were asked to decide which of the presented tones sounds “higher” or “lower” in the tone frequency subtests, and “shorter” or “longer” in the duration subtest, respectively. If the participant’s answer was incorrect, the difference between the paired tones became larger. If the answer was correct, the difference became smaller. This procedure allows for the calculation of individual threshold values based on convergence behavior.

### 2.6. Singing Ability and Singing Behavior during Childhood

We employed a song singing task that had already been used in previous studies [27,35,45,46,48] to assess individual differences in singing ability. Generally, such tasks are laymen (vs. professional musicians) tailored [46,61,62]. Participants are introduced to singing Happy Birthday to the best of their ability in a key to suit their own singing voice. 

The performance assessment was evaluated by singing experts (2 male and 2 female raters). Raters had to score four aspects (melody, vocal range, quality of voice, and rhythm). All four criteria were collapsed into a single score (singing total). Intra-class correlation coefficients were calculated to assess the interrater reliability. Ratings were found to be reliable (see Supplementary Table S3). 

In addition, a multi-item scale concept was used, to obtain participants’ singing behavior during childhood and adolescence. Furthermore, participants reported average hours per week spent singing. Additional instructions were given to ensure that participants referred to the same time period. Childhood was defined as the age-span up to 11 years, and the adolescence period was defined as between 12 and 18 years. It is known that the singing voice reaches approximately two octaves at the age of 10 [63], which is similar to adults without vocal training [36]. The described concepts consist of eight questions each and have been used previously [36,45]. The internal consistencies of the concepts were found to be reliable. Questions and reliability analysis are contained in the Supplementary (see Tables S4 and S5).

### 2.7. Neurophysiological Measurement: Magnetencephalography (MEG)

The Neuromag-122 whole-head MEG system was used to measure the brain responses to seven different sampled instrumental tones (piano, guitar, flute, bass clarinet, trumpet, violin, and drums) and four artificial simple harmonic complex tones. The measurement session duration was 15 min. The procedure is equivalent to previous studies [3,7,8,12,58,64]. The measured auditory evoked fields (AEFs) include the primary auditory P1 response occurring about 30–80 ms after tone onset and the following secondary auditory N1 and P2 responses occurring about 90–250 ms after tone onset. Regarding the temporal dynamics of auditory processing, research identified the first positive auditory evoked response complex (P1) to be one of the most important predictors to explain individual differences in elementary sound perception [3,8]. The following N1 component is proposed to reflect (pre-)attentional processes, sensory stimuli processing [11], and learning-induced plasticity [12], while the P2 response is postulated to be a marker of complex auditory tasks, and sensory integration [13], and it is seen as a biological marker of learning [14]. The primary P1 component and the N1 component are said to be modulated by musical training [3,10], while the P2 response is said to be enhanced after persistent training over longer periods [15].

A bandpass filter of 0.00 (DC)–330 Hz and a sampling rate of 1000 Hz were used to record the AEFs. All 11 tones (instrumental and artificial simple harmonic complex tones) were presented in a pseudorandomized order (tone length 500 ms, inter-stimulus interval range 300–400 ms) and repeated about 100 times. These iterations ensure an optimal signal-to-noise ratio as a prerequisite for robust source modeling enabling analyses of the time course, latencies, and amplitudes of the AEFs. Prior to the MEG recording, subjects’ preparation included the following aspects. The locations of the four head position coils and a set of 35 surface points, including nasion and two pre-auricular points, were determined to identify the participants’ head positions inside the dewar helmet. Participants were introduced to listen passively to the presented sounds. To decrease possible motion artifacts, participants were instructed to listen to the sounds in a relaxed state. Foam earpieces (Ethymotic ER3) were connected via 90 cm plastic tubes (diameter 3 mm) to small, shielded transducers that were fixed in boxes next to the subject’s chair. Stimuli were presented binaurally. The volume was adjusted (70 dB SPL as determined by a Brüel and Kjaer artificial ear (type 4152)). BESA Research 6.0 software (MEGIS Software GmbH, Graefelfing, Germany) was used for data analysis. Before averaging, external artifacts were excluded by applying the BESA research event-related fields (ERF) module. Thus, on average, 3–7 noisy (bad) channels were removed, and around 10% of all epochs exceeding a gradient of 600 fT/cm × s and amplitudes either exceeding 3000 fT/cm or falling below 100 fT/cm were rejected from further analysis. This automatic artifact scan tool removes a substantial part of endogenous artifacts (e.g., eye blinks, eye movements, cardiac activity, face movements, and muscle tensions). A baseline-amplitude calculated over the 100 ms interval before the onset of the tones was subtracted from the signals. The AEFs of each participant were averaged (approximately 1000 artifact-free epochs after the rejection of 10% of artifact afflicted or noisy epochs) using the time window of 100 ms pre-stimulus to 400 ms post-stimulus. Spatio-temporal source modeling was performed applying the spherical head model [65,66] to separate the primary response from the following secondary response complex using the two-dipole model with one equivalent dipole in each hemisphere [10,64]. The P1 wave as a composite response complex comprising separate peaks, including primary and secondary auditory activity, has high inter-individual variability with respect to the shape, the number of sub-peaks, and the timing of peak latencies. Therefore, the fitting interval was adjusted from the peak onset time either toward the saddle point in the case of a two-peak complex or toward the main peak latency in the case of a merged single P1 peak. The P1 response complex undergoes a developmental maturation, while the P1 response complex onset in adults occurs 30–70 ms after tone onset [64]; it can be found in adolescents 40–90 ms and in primary school children 60–110 ms [3,8,11,67] after tone onset. The secondary N1 response typically starts to develop between 8 and 10 years of age [3] and can clearly be separated from the preceding P1 response complex. In a first step, the primary source activity was modeled based on one regional source in each hemisphere using the predefined fitting intervals. In a second step, the location of the fitted regional sources was kept fixed, and then the dipole orientation was fitted towards the highest global field power while keeping its main orientation towards the vertex. Independent of the exact source location in the AC, the high temporal accuracy of P1 peak latency was maintained [12]. The following variables were considered: right and left P1; N1 and P2 peak latencies; and the composite scores for the left and right P1, N1, and P2 latencies (the P1 latency right and left (mean), the N1 latency right and left (mean), and the P2 latency right and left (mean); “mean” indicates the average across hemispheres). In addition, indirect measures of functional lateralization, including the absolute P1 asynchrony |R-L| [P1(Peak){right − left}], the absolute N1 latency asynchrony |R-L| [N1(Peak)(|right − left|)], and the P2 latency asynchrony |R-L| [P2(Peak)(|right − left|)] were considered.

### 2.8. Statistical Analysis

The statistical analyses were performed using the software package IBM SPSS Statistics Version 27.0. In order to contrast the individual differences of the groups, we ran a number of independent *t*-tests for the MEG and the behavioral variables. As a follow-up analysis, we performed two discriminant analyses as this procedure also takes the relationships between variables into account. One discriminant analysis was performed for the MEG variables and another for the behavioral ones. This aimed at illustrating which of the MEG and behavioral variables differentiate our groups best. Finally, in the third step, we performed correlations between the MEG and behavioral variables that separated our groups best, with the aim to show their relationships.

## 3. Results

### 3.1. MEG Variables

**Figure 1:**
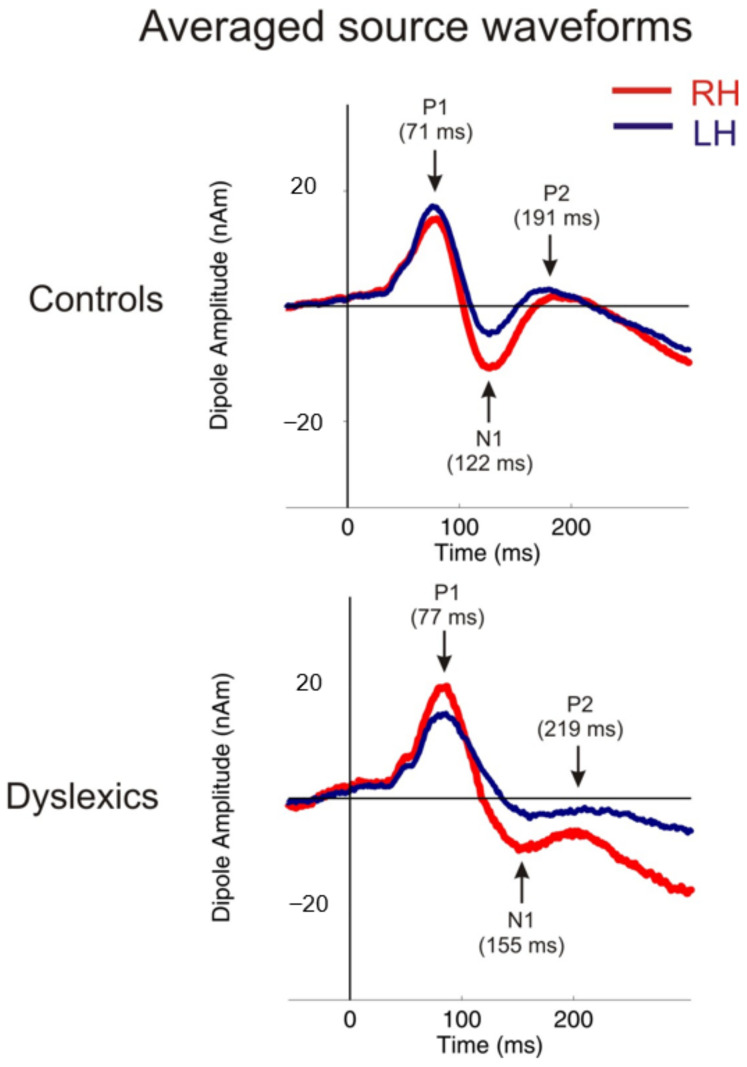
The averaged source waveforms for the dyslexics and controls. The figure demonstrates the averaged source waveforms of the P1–N1–P2 complex in response to various sounds for the right (red) and the left (blue) hemisphere. The controls show a well-balanced hemispheric response pattern, while individuals with dyslexia demonstrate the prolonged response patterns of the entire P1–N1–P2 complex.3.1.1. Independent *t*-tests: MEG.

In the following section, we provide the results of the MEG variables. First, we provide averaged source waveforms for dyslexics and controls in Figure 1, followed by independent *t*-tests and a discriminant analysis. Individuals with dyslexia showed prolonged latencies in the composite scores for the left and right P1, and N1 and P2, latencies (see Table 1 and Table 2).

#### 3.1.1. Independent *t*-Tests: MEG 

Table 1 below shows the independent *t*-tests and the descriptives of the MEG variables of the controls and the individuals with diagnosed dyslexia. In order to avoid an accumulation of the alpha error for multiple testing, we applied a Benjamini–Hochberg correction. Descriptions of the MEG variables are provided in Section 2.8.

**Table 1:** The independent *t*-tests and the descriptives of the MEG variables.

Variables	Controls: Mean ± SE	Controls: Min.|Max.	Dyslexia: Mean ± SE	Dyslexia: Min.|Max.	*p*	*r*
P1 latency right and left (mean) +	70.6 4 ± 1.94	56.50|97.50	77.71 ± 2.75	59.50|101.00	*p* < 0.041	*r* = 0.29
absolute P1 latency asynchrony |R-L|	3.89 ± 0.75	0.00|13.00	8.65 ± 2.66	1.00|70.00	*p* < 0.091	*r* = 0.24
N1 latency right and left (mean) +	121.52 ± 2.38	104.00|163.00	154.58 ± 9.18	106.50|236.50	*p* < 0.001	*r* = 0.44
absolute N1 latency asynchrony |R-L|	9.62 ± 2.53	0.00|52.00	16.85 ± 6.16	4.00|141.00	*p* < 0.283	*r* = 0.15
P2 latency right and left (mean) +	191.15 ± 6.29	144.00|251.50	219.50 ± 12.31	144.50|357.00	*p* < 0.046	*r* = 0.28
absolute P2 latency asynchrony |R-L|	11.81 ± 2.37	0.20|28.80	21.27 ± 4.56	0.10|31.20	*p* < 0.071	*r* = 0.25

+ indicates that the *t*-test remains significant after Benjamini–Hochberg correction for multiple testing (*p* < 0.05).

#### 3.1.2. Discriminant Analysis: MEG

The results of the discriminant function revealed that the P1, N1, and P2 latencies separated the dyslexics from the control group best, Λ = 0.69, χ^2^(6) = 17.42, *p* < 0.008, and the canonical R2 = 0.31. The correlations between the MEG variables and the discriminant function revealed that the following variables: N1 latency right and left (*r* = 0.74), P1 latency right and left (*r* = 0.44), and P2 latency right and left (*r* = 0.43), which showed loads above the statistical acceptable cut off value of 0.4 [68] on the discriminant function. Table 2 below illustrates the structure matrix of the MEG variables and illustrates the loads of the MEG predictors on the discriminant function.

**Table 2:** The discriminant function of the MEG variables. The table shows the correlations of the outcome variables and the discriminant function of the MEG variables. We used the statistically accepted cutoff value of 0.40 to decide which of the variables were large enough to discriminate between the groups (see bold numbers).

	MEG Predictors
*N1 latency right and left (mean)*	**0.736**
*P1 latency right and left (mean)*	**0.444**
*P2 latency right and left (mean)*	**0.433**
*absolute P2 latency asynchrony |R-L|*	0.389
*absolute P1 latency asynchrony |R-L|*	0.364
*absolute P2 latency asynchrony |R-L|*	0.229

### 3.2. Behavioral Variables

#### 3.2.1. Independent *t*-Tests: Behavioral

Table 3 below shows the independent *t*-tests of the music and language variables of the controls and the individuals with diagnosed dyslexia. The results revealed that the control group performed significantly better than the individuals with dyslexia in all the Mandarin subtests, in the music measures, and in the elementary auditory skills except for duration (see Table 3). Singing hours per week, singing behavior during childhood, and singing behavior during adolescence are not provided in Table 3 since they failed to be significantly different across groups, even if we had decided to use a one tailed approach (see Supplementary Table S7). In order to avoid an accumulation of the alpha error for multiple testing, we applied a Benjamini–Hochberg correction. The independent *t*-tests and the descriptives for the subscores of the music and the singing variables are contained in the Supplementary (Tables S6 and S7).

#### 3.2.2. Discriminant Analysis: Behavioral

The results of the discriminant function revealed that most of our music and language variables separated the groups well, Λ = 0.42, χ^2^(7) = 40.02, *p* < 0.001, and the canonical R2 = 0.58. The correlations between the considered variables and the discriminant function revealed that tone frequency showed the highest load on the discriminant function, followed by Mandarin P, singing total, AMMA total, and Mandarin S, which were all above the statistical acceptable cut off value of 0.4 [68]. Mandarin D and duration failed to discriminate the groups and were below the statistical acceptable cut off value. Table 4 below illustrates the structure matrix of the behavioral predictor variables and the loads of each predictor variable on the discriminant function.

### 3.3. Correlational Analysis

**Figure 2:**
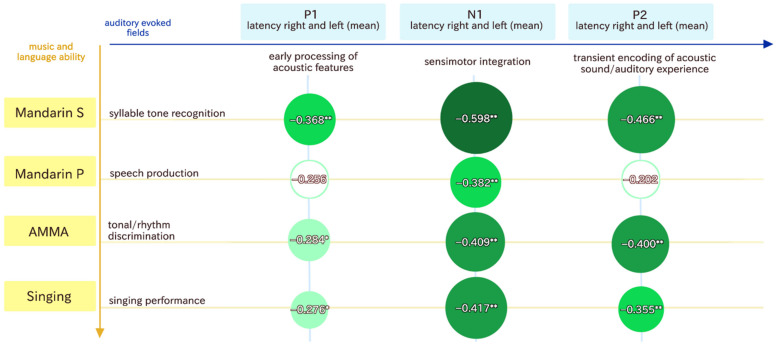
This figure shows the correlations between the P1, N1, and P2 responses and the behavioral variables. * means significant at the 0.05 level (2-tailed), and ** means that correlations are significant at the 0.01 level. The color green represents the effect size of the correlation coefficient. The darker the green, the larger the effect.

The two discriminant functions were the basis for the follow-up correlational analysis. Therefore, we used the variables that differentiated between our groups (controls versus dyslexia) best and correlated them in order to outline which of the MEG variables are associated with the music and language variables. Figure 2 shows the most important behavioral measures, which also correlated with the MEG variables. A correlational analysis between the MEG and behavioral variables revealed that Mandarin syllable tone recognition, singing ability, and musical aptitude (AMMA) correlated with the P1, N1, and P2 latencies, while Mandarin pronunciation was only associated with N1 latency.

## 4. Discussion

We assessed the language ability in Mandarin, the ability to sing, the musical talent, the individual differences in elementary auditory skills, and the auditory evoked the P1–N1–P2 complex of 52 adolescents and young adults with dyslexia and unaffected controls. The analysis revealed that most music and language measures differed significantly between dyslexics and controls. The discriminant analysis showed that individuals with dyslexia performed lower than controls in discriminating high versus low tones (frequency), in musical ability tasks, in singing, and in Mandarin pronunciation and the sound-syllable tone recognition task. In addition, the discriminant analysis for the auditory-evoked field variables revealed that individuals with dyslexia showed prolonged latency (right and left mean) of the P1, N1, and P2 response components. As a follow-up, correlations between the behavioral and the MEG variables were performed in order to uncover whether they are interrelated. Correlational analysis revealed that tone frequency was the only behavioral variable that did not correlate with any of the MEG variables. In the following sections, we discuss the P1, N1, and P2 responses and their correlations with the behavioral variables.

In general, the primary and secondary auditory cortex are the areas from which the P1 and N1 components of the event-related fields are said to emerge [69]. Both P1 and N1 represent early feature processing, with the P1 response being known to occur around 50–80 ms after tone onsets in adolescents [15] and the N1 response after around 80–110 ms [70]. The following P2 response is seen about 160–200 ms after tone onset [15]. The P1 complex has been associated with the ability to perceive auditory stimuli [3,8,10]. It can already be detected in early childhood [71]. While the individual differences of the P1 complex can already be measured in childhood, the N1 response usually emerges later, between 8 and 10 years of age [67,71]. The N1 response is presumed to be an intermediate stage in auditory analysis contributing to sound detection [72]. Further, the N1 response reflects integrative processes and shows a strong context dependency and learning-induced plasticity [12]. Additionally, studies suggested that the N1 response component reflects sensory stimuli processing and sensorimotor integration [70,73], as well as attention-specific processes [74]. The following P2 response has been postulated to be sensitive to auditory experience [75] and seems to be a marker of complex auditory task processing such as melody and rhythm recognition. However, the P2 complex has also been associated with sensory integration [13] and sensory processes, such as the transient encoding of acoustic sound features, as well as those involved in forming short-term sound representation in sensory memory [72]. In addition, the P2 complex has also been defined as a biological marker of learning [75].

There is evidence from previous studies that the P1 source waveform responses are well-balanced in controls, whereas individuals with dyslexia show prolonged P1 [7,8] and N1 latencies [7]. Beside prolonged P1 and P2 latencies, the P2 response was shown to be larger in the participants who were diagnosed dyslexia. In general, reduced latencies are associated with higher attention, and faster and more precise auditory processing [7,8,76]. Considering that N1 and P2 responses are also associated with sensory processing, reduced latency can also be understood as an enhanced sensory processing skill. This claim can also be supported by language disorder frameworks, which suggest that dyslexia is a deficit of impaired auditory sensory integration of incoming acoustic signals [77,78]. In former studies, we used various musical measures to uncover relationships between auditory processing and musical ability. Findings indicated that individuals with dyslexia showed impairments in elementary (e.g., frequency and duration) and complex auditory sound discrimination (e.g., AMMA measures) [3]. These findings were linked to deficits in P1 responses. In a follow-up study, we noted that lower musical performance (e.g., playing an instrument or singing) was also associated with prolonged N1 latencies. Therefore, we presume that the lower musical performances of patients with dyslexia could also be understood as auditory sensory impairment [7]. 

The results of our behavioral measures provide further evidence that dyslexia might be a disorder featuring auditory and sensory deficits. Thereby, individuals with dyslexia performed worse in music and language measures regardless of whether they were classified as perception or production variables. The discriminant analysis revealed that the sound-symbol correspondence task (Mandarin S), Mandarin pronunciation, and singing ability and musical ability (AMMA) were the most important behavioral variables separating the groups best. The latter, musical ability, has already been investigated in detail. It is understood that the P1, N1, and subsequent P2 responses are modulated by musical input and training, including singing [3,10,79]. The P1 and N1 responses were prolonged and faster in musicians [3,80], and the P2 response complex was considerably enlarged as a result of musical long-term experience [79], which underlines that musical training modulates the entire P1–N1–P2 complex. 

The Mandarin S task requires individuals to detect a tonal change in the second statement. This includes that more than one process takes place at the same time. First, they have to recognize a tonal change and second link the tonal change to a specific syllable—an ability that is linked to individual differences in reading and writing skills. In particular, this task can be considered to be a sound-symbol correspondence task and has a strong musical component in which individuals have to recognize a change of the tone syllable. In this respect, this task requires the precise ability to discriminate between auditory stimuli and high attentional skills and is more complex than, for instance, language pronunciation tasks. This may be one possible explanation for why we detected that the Mandarin S task is negatively correlated with the P1, N1, and P2 responses, which means that the lower the latencies, the better the Mandarin S performance. The Mandarin pronunciation task is indeed simpler than the Mandarin S task and is of high ecological validity as it simulates a situation in which new languages are acquired [36,45,46]. Therefore, Mandarin pronunciation tasks provide information about phonological skills and the ability to imitate new words. Pronunciation tasks such as our Mandarin task, as well as their relationship to musical ability across all ages, have often been employed in language aptitude research [30,36,45,46]. Musical ability, particularly singing ability, is related to the ability to pronounce, retrieve, and imitate new language material. Behavioral research suggests that enhanced vocal flexibility [27], vocal control [28,35], and vocal-motor and auditory-motor integration [81,82] are some of the underlying mechanisms that explain their close relationship. 

In consideration of the findings of this study, the results of the aforementioned behavioral studies are supported by the MEG results. Both the ability to sing and to pronounce Mandarin were negatively correlated to the N1 latency, meaning the lower the latency, the better the singing and pronunciation performances. The N1 response component reflects sensory stimuli processing and sensorimotor integration [70,73]. Therefore, this study provides additional evidence that sensory ability is the reason why singing and the ability to learn to pronounce new languages have been found to be very interrelated in behavioral testing [27,30,35]. 

We are aware of several limitations in our study. Hence, future studies should include larger sample sizes and outline whether similar or distinct findings will be made when EEG recording is conducted.

## 5. Implications and Future Research Directions

Music and language exhibit overlaps on multiple dimensions. In this study, we provided additional evidence that language and music processing are interrelated in the auditory cortex. This supports the commonly accepted frameworks on music and language relationships such as the OPERA hypothesis [37], which proposes that musical training and ability improves language capacity. 

Former studies provided evidence that professional musicians outperformed non-musicians in detecting Mandarin syllable discrimination tasks and in the ability to pronounce Mandarin [53]. Musical training studies made similar observations and have outlined that pitch processing in Mandarin improves through the playing of instruments [83]. Consequently, the acquisition of Mandarin tone syllables requires the precise ability to process tonal material as an additional cognitive capacity. This may be a further aspect that should be addressed when individuals with dyslexia acquire tone languages, and we suggest that at least two crucial aspects have to be addressed in future studies. First, such studies should assess whether musical training may be a beneficial tool to support individuals with dyslexia when they learn a tone language. Second, they should consider that learning Mandarin requires being able to link syllable tones to Mandarin orthography—a rather complex process. Research also noted that the orthographic complexity of specific languages exacerbates the symptoms of dyslexia [84], which is why future research should also study dyslexia and Mandarin in more detail. Our findings suggest that dyslexics also have deficits in sensory stimuli processing and the sensorimotor integration of vocalization regardless of whether they sing or imitate new languages. Various therapies have been developed in which singing plays a major role in improving fluency in speech. For instance, (intoned) singing is part of *Melodic Intonation Therapy* and aims to improve the spontaneous speech production in patients who suffer from non-fluent aphasia [85]. Singing has also been employed as a tool to reduce stuttering and to gain better vocal motor control [86]. However, we want to note that singing facilitates pronunciation skills in general. Therefore, singing should not be used to learn syllable tones in Mandarin as the latter become neutralized when they are sung [53]. Moreover, we propose singing to be a good tool to train self-monitoring, to integrate vocal tract-related skills, and to develop sensory motor representations in order to gain better vocal motor control while articulating [87]. This suggests that future research directions should investigate whether singing would be a useful tool to improve the ability to learn new words in individuals who are diagnosed with dyslexia.

## Figures and Tables

**Table 3:** The independent *t*-tests and the descriptives of the variables of the investigation.

Variables	Controls: Mean ± SE	Controls: Min.|Max.	Dyslexia: Mean ± SE	Dyslexia: Min.|Max.	*p*	*r*
Mandarin D: Tone discrimination +	6.27 ± 1.71	2.00|8.00	5.23 ± 1.42	3.00|8.00	*p* < 0.021	*r* = 0.32
Mandarin S: Syllable tone recognition +	7.19 ± 0.32	4.00|10.00	5.46 ± 0.39	1.00|9.00	*p* < 0.001	*r* = 0.43
Mandarin P +	4.50 ± 0.22	2.34|6.03	3.26 ± 0.20	1.71|5.64	*p* < 0.000	*r* = 0.51
Singing total +	6.47 ± 0.22	4.19|8.81	5.38 ± 0.17	3.81|6.81	*p* < 0.000	*r* = 0.49
AMMA total +	52.77 ± 1.53	41.00|76.00	44.69 ± 1.48	30.00|58.00	*p* < 0.000	*r* = 0.47
Duration	39.78 ± 3.78	9.50|107.70	50.04 ± 4.67	15.70|93.80	*p* < 0.094	*r* = 0.26
Tone Frequency +	14.87 ± 2.68	1.30|70.80	45.29 ± 5.30	5.10|104.60	*p* < 0.000	*r* = 0.55

+ indicates that the *t*-test remains significant after Benjamini–Hochberg correction for multiple testing (*p* < 0.05). The following description explains the acronym definitions of the variables. Mandarin D: tone discrimination task; Mandarin S: syllable tone discrimination task; Mandarin P: pronunciation task; the singing total consists of the subscores singing melody, singing vocal range, singing voice quality, and singing rhythm; AMMA total: Advanced Measures of Music Audiation—the total score consisting of rhythmic and tonal subtests; duration: the primary auditory threshold test—subtest duration; tone frequency: the primary auditory threshold test—subtest frequency.

**Table 4:** The discriminant function of the behavioral variables. The table shows the correlations of the outcome variables and the discriminant function of the behavioral predictor variables. We used the statistically accepted cutoff value of 0.40 to decide which of the variables were large enough to discriminate between the groups.

	Behavioral Predictors
*Tone frequency*	−0.621
*Mandarin P*	0.537
*Singing total*	0.506
*AMMA total*	0.482
*Mandarin S: Syllable tone recognition*	0.432
*Mandarin D: Tone discrimination*	0.302
*Duration*	−0.217

## Data Availability

Data are contained within the article or Supplementary Materials.

## Supplementary Materials

The following supporting information can be downloaded at: https://www.mdpi.com/article/10.3390/brainsci12060744/s1, Table S1: Description of participants; Table S2: Mandarin intraclass coefficients; Table S3: singing intraclass coefficients; Table S4: Cronbach’s α coefficients for the singing during childhood questionnaire; Table S5: Cronbach’s α coefficients for the singing during adolescent questionnaire; Table S6: Independent *t*-tests and descriptives of the musical sub-variables; Table S7: Independent *t*-tests and descriptives of the singing sub-variables.
