# Supplementary material for: Examining Individual Differences in Singing, Musical and Tone Language Ability in Adolescents and Young Adults with Dyslexia
Source: Brain Sci. 2022 Jun 6;12(6):744. doi: 10.3390/brainsci12060744 (PMC9221489; doi:10.3390/brainsci12060744)
Supplement: Supplementary file 1 [file brainsci-12-00744-s001.zip › brainsci-1744807-supplementary.pdf]

brain sciences

MDPI

Article

Supplementary Materials: The following supporting information can be downloaded at: https://www.mdpi.com/article/10.3390/brainsci12060744/s1, Table S1: Description of participants; Table S2: Mandarin intraclass coefficients; Table S3: singing intraclass coefficients; Table S4: Cronbach's α coefficients for the singing during childhood questionnaire; Table S5: Cronbach's α coefficients for the singing during adolescent questionnaire; Table S6: Independent *t*-tests and descriptives of the musical sub-variables; Table S7: Independent *t*-tests and descriptives of the singing sub-variables

Description of participants

Table S1. Description of participants.

Parameters	Categories	Dyslexia	Control
**N**		26	26
**Age**	*Mean ± SD*	16.88 ± 1.31	17.73 ± 0.92
**Sex**	*female*	9	14
	male	17	12
**Education**	*Main general secondary school*	17 (65.4%)	18 (69.2%)
	Secondary academic school	9 (34.6%)	8 (30.8%)

Note that for the educational background the participants were instructed to indicate their school-leaving qualifications they had already achieved during testing time.

Intraclass coefficients

The tables included below report the reliability analyses we performed for the variables included in this study. Specifically, we computed intraclass correlation coefficients on the ratings for participants' performances on the Mandarin pronunciation by using a two-way mixed effects models where people effects were random and measures effects were fixed. For the Mandarin performances we had five raters for the tasks which consisted of seven and eleven syllables and four raters for the Mandarin sample which consisted of nine syllables. The same procedure was performed for the singing ratings. For the singing ratings we had four singing teachers who rated each of the four singing criteria (voice quality, rhythm, melody and vocal range). The results have shown that the ratings were reliable and above the accepted value of 0.7 [27]. Table S1 shows the Mandarin intraclass coefficients, while table S2shows the singing intraclass coefficients.

Table S2. Mandarin intraclass coefficients.

	Average Measures	*95% Confidence Interval*		*F Test with True Value 0*			
	Intraclass Correlation	Lower Bound	Upper Bound	Value	df1	df2	Sig
Mandarin seven syllables	0.83	0.68	0.91	9.26	51	204	0.000

Mandarin nine syllables	0.83	0.68	0.91	9.26	51	204	0.000
Mandarin eleven syllables	0.74	0.58	0.85	5.17	51	204	0.000

Table S3. Singing intraclass coefficients.

	Average Measures	*95% Confidence Interval*		*F Test with True Value 0*			
	Intraclass Correlation	Lower Bound	Upper Bound	Value	df1	df2	Sig
Singing melody	0.86	0.77	0.92	8.46	51	153	0.000
Singing vocal range	0.84	0.69	0.91	8.89	51	153	0.000
Singing quality of voice	0.86	0.79	0.91	8.56	51	153	0.000
Singing rhythm	0.77	0.61	0.86	5.43	51	153	0.000

Singing behavior during childhood and adolescence

To assess the interrater reliability of the individual eight questions asking participants about their singing behavior during childhood and singing behavior during adolescence, we computed Cronbach's α coefficients. The results (Tables S4 and S5) show that the reliability for both concepts is high, with all Cronbach's α =74 for singing behavior during childhood and with all, Cronbach's α =77 for singing behavior during adolescence. Thus, the overall value above the statistically accepted range of 0.7. Tables S3 and S4 below show the individual contribution of each question to the multi-item scale concepts. The questions are translations since the original questionnaire was written and assessed in German.

Table S4. Cronbach's α coefficients for the singing during childhood questionnaire.

Concept *Singing behavior during childhood (6-11 years)*

	Cronbach's α if item deleted
As a child I enthusiastically joined in with the singing at church and similar events whenever the possibility arose (Q1).	0.707
As a child I was encouraged to sing by my caretakers and we sang together on a weekly basis even if there were no special events. (Q2).	0.744
As a child I enjoyed singing in a choir, with friends, at Christmas, birthdays, or at similar occasions (Q3).	0.681
As a child I sang very often since I wanted to become a musician or singer (Q4).	0.728
As a child I used to sing whenever I could such as in the bathroom, in the car, when I played with friends (Q5).	0.676
As a child I liked being a member of our school choir, or would have liked being a member or a school choir (Q6).	0.661
As a child I enjoyed singing a song that had been played to me (e.g., in the radio) (Q7).	0.718
As a child I used to sing more often than my friends (Q8).	0.748

Table S5. Cronbach's α coefficients for the singing during adolescent questionnaire.

Concept *Singing behavior during adolescent (12-18 years)*

	Cronbach's α if item deleted
As an adolescent I enthusiastically joined in with the singing at church and similar events whenever the possibility arose (Q1).	0.722
When I was an adolescent, I didn't think about anything when I sang. I just started singing. (Q2).	0.781

As an adolescent I enjoyed singing in a choir, with friends, at Christmas, birthdays, or at similar occasions (Q3).	0.690
As an adolescent I sang very often since I wanted to become a musician or singer (Q4).	0.771
When I was an adolescent, I sang whenever I could. (Q5).	0.724
As an adolescent I liked being or would have liked being a member of a music band (Q6).	0.686
As an adolescent I enjoyed singing a song that had been played to me (e.g., in the radio) (Q7).	0.818
As an adolescent I used to sing more often than my friends (Q8).	0.707

Independent *t*-tests

We also assessed the individual subscores of our music measurements which are described in sections 2.5 and 2.7. in the manuscript. The results are presented in tables S6 and S7 below. In order to avoid an accumulation of the alpha error for multiple testing we applied a Benjamini-Hochberg correction. While table S56shows the music perception subscores, table S7 illustrates the singing subscores of this investigation.

Table S6. Independent *t*-tests and descriptives of the musical variables.

**variables**	**controls: mean** ± SE	**controls: min.**|**max.**	**dyslexia: mean** ± SE	**dyslexia: min.**|**max.**	***p***	***r***
AMMA T +	24.96 ± 0.79	17|39	21.65 ± 0.73	16|29	$p < 0.003$	$r = 0.47$
AMMA R +	27.81 ± 0.82	20|37	23.04 ± 0.86	14|31	$p < 0.000$	$r = 0.40$

+ remain significant after Benjamini-Hochberg correction for multiple testing ($p < 0.05$). The following description explains the acronym definitions of the variables. AMMA R: Advanced Measures of Music Audiation – rhythmic score; AMMA T: Advanced Measures of Music Audiation – tonal score

Table S7. Independent *t*-tests and descriptives of the singing variables.

**variables**	**controls: mean** ± SE	**controls: min.**|**max.**	**dyslexia: mean** ± SE	**dyslexia: min.**|**max.**	***p***	***r***
Singing behavior during childhood	43.15 ± 2.94	18|70	38.50 ± 2.33	16|72	$p < 0.221$	$r = 0.17$
Singing behavior during adolescence	36.85 ± 3.05	17|67	30.62 ± 2.44	12|51	$p < 0.117$	$r = 0.22$
Singing hours per week	1.98 ± 0.44	0|10	1.04 ± 0.39	0|10	$p < 0.118$	$r = 0.22$
Singing melody +	6.74 ± 0.28	3.5|9.5	5.38 ± 0.17	3.5|7.5	$p < 0.000$	$r = 0.53$
Singing vocal range +	6.56 ± 0.29	3.75|8.75	5.40 ± 0.24	3.25|8.0	$p < 0.004$	$r = 0.40$
Singing quality of voice +	6.26 ± 0.20	4.25|8.75	5.56 ± 0.16	4.0|7.25	$p < 0.009$	$r = 0.36$
Singing rhythm +	6.33 ± 0.16	4.75|8.25	5.35 ± 0.15	3.75|6.75	$p < 0.000$	$r = 0.54$

+ remain significant after Benjamini-Hochberg correction for multiple testing ($p < 0.05$).
